# Efficacy of Artemisinin-Based Combination Treatments of Uncomplicated Falciparum Malaria in Under-Five-Year-Old Nigerian Children

**DOI:** 10.4269/ajtmh.13-0248

**Published:** 2014-11-05

**Authors:** Stephen Oguche, Henrietta U. Okafor, Ismaila Watila, Martin Meremikwu, Philip Agomo, William Ogala, Chimere Agomo, Godwin Ntadom, Olajide Banjo, Titilope Okuboyejo, Gboye Ogunrinde, Friday Odey, Olugbemiga Aina, Tolulope Sofola, Akintunde Sowunmi

**Affiliations:** Antimalarial Therapeutic Efficacy Monitoring Group, The Federal Ministry of Health, Abuja, Nigeria

## Abstract

The efficacy of 3-day regimens of artemether-lumefantrine and artesunate-amodiaquine were evaluated in 747 children < 5 years of age with uncomplicated malaria from six geographical areas of Nigeria. Fever clearance was significantly faster (*P* = 0.006) and the proportion of children with parasitemia 1 day after treatment began was significantly lower (*P* = 0.016) in artesunate-amodiaquine—compared with artemether-lumefantrine-treated children. Parasite clearance times were similar with both treatments. Overall efficacy was 96.3% (95% confidence interval [CI] 94.5–97.6%), and was similar for both regimens. Polymerase chain reaction-corrected parasitologic cure rates on Day 28 were 96.9% (95% CI 93.9–98.2%) and 98.3% (95% CI 96.1–99.3%) for artemether-lumefantrine and artesunate-amodiaquine, respectively. Gametocyte carriage post treatment was significantly lower than pretreatment (*P* < 0.0001). In anemic children, mean time to recovery from anemia was 10 days (95% CI 9.04–10.9) and was similar for both regimens. Both treatments were well tolerated and are safe and efficacious treatments of uncomplicated falciparum malaria in young Nigerian children.

## Introduction

Artemisinin-based combination treatments (ACTs), the recommended first-line treatments of uncomplicated falciparum malaria globally,^1^ have been adopted by most endemic countries.^2^ However, recent reports of resistance to artemisinin in *Plasmodium falciparum* in some parts of Southeast Asia where ACTs have been deployed for over a decade as first-line treatments,^3^–^7^ has made it necessary to continue surveillance of the efficacy of ACTs, especially in areas where artemisinin resistance in *P. falciparum* has not yet been reported. Historically, resistance to antimalarial drugs originates from Southeast Asia and South America, and then spreads to East and Central Africa, and to West Africa.^8^ In Africa, resistance first becomes evident in children < 5 years of age because of a relative lack of antimalarial immunity.^9^

In Nigeria, artemether-lumefantrine and artesunate-amodiaquine were adopted as first-line treatments in 2005.^10^ However, after 5 years of adoption as first-line treatments there is no study of their efficacy in all geographical areas of the country. To detect early changes in responses of *P*. *falciparum* to artemether-lumefantrine and artesunate-amodiaquine, we evaluated the therapeutic efficacy of artemether-lumefantrine and artesunate-amodiaquine in Nigerian children < five years of age. The primary aims were to compare the efficacy of these ACTs in all malaria-endemic settings in Nigeria and to assess changes in *P. falciparum* susceptibility to ACTs.

## Materials and Methods

### Study locations.

**Figure 1.**
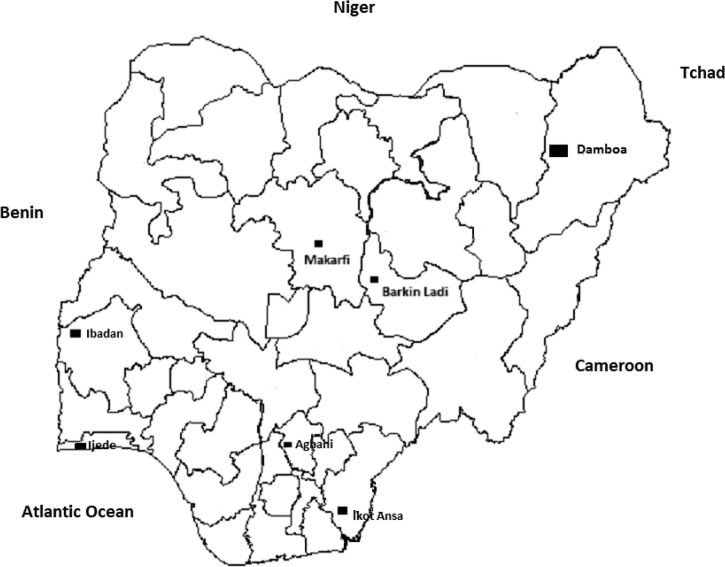
Map of Nigeria showing study locations in seven states.

This study was an open-label study trial carried out between October 2009 and November 2010 at the following locations: Agbani, Ikot Ansa, Barkin Ladi, and Damboa, in Enugu, Cross River, Plateau, and Borno states, respectively (the eastern flank of the study sites), and in Ijede, Sabo quarters of Ibadan and Makarfi in Lagos, Oyo, and Kaduna states, respectively (the western flank) (Figure 1). In virtually all study locations, malaria is hyperendemic and transmission occurs all year round; however, it is more intense during the rainy season from April to October.

### Patients, treatments, and follow-up.

Patients were eligible to join the study if they were < 5 years of age, had symptoms compatible with acute uncomplicated falciparum malaria such as fever, anorexia, vomiting, or abdominal discomfort with or without diarrhea, with *P. falciparum* parasitemia between 1,000 and 200,000 asexual forms per μL, a body (axillary) temperature > 37.4°C, or history of fever in the 24 to 48 h preceding presentation, absence of other concomitant illness, no history of antimalarial use in the 2 weeks preceding presentation, and written informed consent given by parents or guardians. Patients with severe malaria,^11^ severe malnutrition, serious underlying diseases (renal, cardiac, or hepatic), and known allergy to study drugs were excluded from the study. The study protocol was approved by National Health Research Ethics Committee, Abuja, Nigeria. The disease history, taken by the attending physician, was recorded by asking parents/guardians when the present symptomatic period started and was followed by a full physical examination by the same physician.

Enrolled patients were randomized to receive artemether-lumefantrine or artesunate-amodiaquine (co-formulated) given according to body weight. Artemether-lumefantrine (Coartem, Novatis, Basel, Switzerland) was given as follows: patients weighing 5–14 kg received one tablet, and those weighing 15–24 kg received two tablets at presentation (0 hour), 8 hours later, and at 24, 36, 48, and 60 hours after the first dose. Each tablet of artemether-lumefantrine contains 20 mg of artemether and 120 mg of lumefantrine. Artesunate-amodiaquine (Coarsucam, Sanofi Aventis, France) was also given as follows: patients weighing ≥ 4.5 to < 9 kg received one tablet, those weighing ≥ 9 to < 18 kg received one tablet, and those weighing ≥ 18 to < 24 kg received one tablet of the following formulations: 25 mg/67.5 mg, 50 mg/135 mg, 100 mg/270 mg of fixed dose combination of artesunate/amodiaquine, respectively, daily for 3 days.

All drugs were given orally. In patients treated with artesunate-amodiaquine, the drug was given as single day or single daily doses in the clinic by the physician. In patients treated with artemether-lumefantrine, the 0-, 24-, and 48-hour doses were given in the clinic by the physician, and the 8-, 36-, and 60-hour doses were given by parents or guardians of the children at home. Parents or guardians were questioned at follow-up on the time and events after drug administration. After drug administration in the clinic, all patients waited for at least 30 minutes to ensure the drug was not vomited. If it was, the dose was repeated. If the repeat dose was vomited, the patient was excluded from the study. If necessary, patients were provided with antipyretics (paracetamol tablets 10–15 mg/kg every 8 hours for 24 hours). The randomization was computer generated and treatment codes were sealed in individual envelopes. Patient evaluation and follow-up after administration was performed by another physician blinded to the drug treatment. Thick and thin blood films were obtained from each child as soon as they came to the clinic and the slides were carefully labeled with the patients' codes and air-dried before being stained.

Follow-up with clinical and parasitological evaluation was done daily on Days 1–3 and on Days 7, 14, 21, and 28. This consists of enquiry about the patients well being, presence or absence of initial presenting symptoms, presence of additional symptoms, measurement of body temperature, heart and respiratory rates, and a blood smear for quantification of parasitemia.

Side effects were defined as symptoms and signs that first occurred or became worse after treatment was started and were checked for at every visit. Laboratory tests to further elicit adverse events were not performed routinely at every visit. Any new events occurring during treatment were also considered as side effects.

Thick and thin blood films prepared from a finger prick were stained with Giemsa and examined by light microscopy under an oil-immersion objective at 1,000× magnification by two independent assessors who did not know the drug regimen of the patients. A senior member of the study team reviewed the slides if there was any disagreement by the two microscopists. In addition, the slides of every fourth child enrolled in the study were reviewed by the senior member. Parasitemia, asexual or sexual, in thick films were estimated by counting asexual and sexual parasites relative to 500 leukocytes, or 500 asexual or sexual forms, whichever occurred first. From this figure, the parasite density was calculated assuming a leukocyte count of 6,000/μL of blood. A slide was considered parasite negative if no asexual or sexual parasite was detected after examination of 200 microscope fields.

Capillary blood, collected before and during follow-up, was used to measure packed cell volume (PCV). The PCVs were measured using a microhematocrit tube and microcentrifuge (Hawksley, Lancing, UK).

Blood was spotted on filter paper on Days 0, 3, 7, 14, 21, and 28, and at the time of treatment failures for parasite genotyping. Paired primary and post treatment samples were analyzed using parasites' polymorphic genes to distinguish recrudescence from new infections. Briefly, block 2 of merozoite surface protein-1 (MSP-1) and the block 3 of merozoite surface protein-2 (MSP-2) and region II of glutamine-rich protein (GLURP) were amplified by two rounds of polymerase chain reaction (PCR) using specific primers as previously described.^12^–^16^ Ten microliters of the nested PCR product were resolved by electrophoresis on a 2% agarose gel and sized against 100-basepair molecular mass marker (New England Biolabs, Beverly, MA).

The banding pattern of post-treatment parasite was compared with matched primary parasites in each of the patients who had parasitemia after treatment with either artemether-lumefantrine or artesunate-amodiaquine. Post treatment and primary infection parasites showing identical bands were considered as recrudescence, whereas non-identity in banding patterns was considered as newly acquired infections. To confirm the absence of recurrent parasitemia, samples obtained in one in every four patients with microscopically negative blood films were also subjected to PCR analysis.

Response to drug treatment was assessed using a modified version of the World Health Organization (WHO) 2003 *in vivo* clinical classification criteria.^9^ Because all patients were not febrile at enrollment, a temperature < 37.5°C was not an exclusion criterion for enrollment. The modification also involved a follow-up for 28 days in these areas of intense transmission. The clinical classification system consisted of the following categories of response: adequate clinical and parasitological response (ACPR), late parasitologic failure (LPF), late clinical failure (LCF), and early treatment failure (ETF). The primary outcomes were the 28-day uncorrected and PCR-corrected efficacy. The secondary outcomes were the fever and parasite clearance times, gametocyte carriage, and recovery from malaria-associated anemia.

Cure rates were defined as the percentages of patients whose asexual parasitemia cleared from peripheral blood and who were free of patent asexual parasitemia on Days 14, 21, and 28 of follow-up. The cure rates on Days 21 and 28 were adjusted on the basis of the PCR genotyping results of paired samples of patients with recurrent parasitemia after Day 7 of starting treatment.

### Retreatment of drug treatment failures.

All patients failing treatment, defined as reappearance of parasitemia after its initial complete clearance or failure of complete clearance within 28 days, were retreated whether they became symptomatic or not and after samples had been obtained for microscopy and PCR analysis. Patients failing treatment with artemether-lumefantrine were retreated with artesunate-amodiaquine and vice versa, and were followed up for another 28 days.

### Data analysis.

Sample size was calculated so that the study will be able to detect a 5% absolute difference in parasitologic failure rate between artemether-lumefantrine and artesunate-amodiaquine treatment groups with 95% power and at a 5% significance level. The expected treatment success rates were 100% for artemether-lumefantrine and 95% for artesunate-amodiaquine on Day 28. Except at Ikot Ansa, where a total of 63 children was enrolled in the two treatment arms, a minimum of 50 patients per treatment arm were enrolled at each site. Data were analyzed using version 6 of Epi-Info software^17^ and the statistical program SPSS for Windows version 17.0.^18^ Variables considered in the analysis were related to the densities of *P. falciparum* asexual and sexual forms. Proportions were compared by calculating χ^2^ using Yates' correction, Fisher's exact or Mantel Haenszel tests. Normally distributed, continuous data were compared by Student's *t* test and analysis of variance. Data not conforming to a normal distribution were compared by the Mann-Whitney *U* tests and the Kruskal Wallis tests (or by Wilcoxon ranked sum test). The risk for parasite reappearance was calculated by survival using the Kaplan-Meier method. *In vivo* parasitological measures of artemisinin susceptibility were calculated as previously described.^19^ The *P* values of ≤ 0.05 were taken to indicate significant differences. Data were double entered serially using patients' codes and were only analyzed at the end of the study.

## Results

### Patient characteristics.

**Figure 2.**
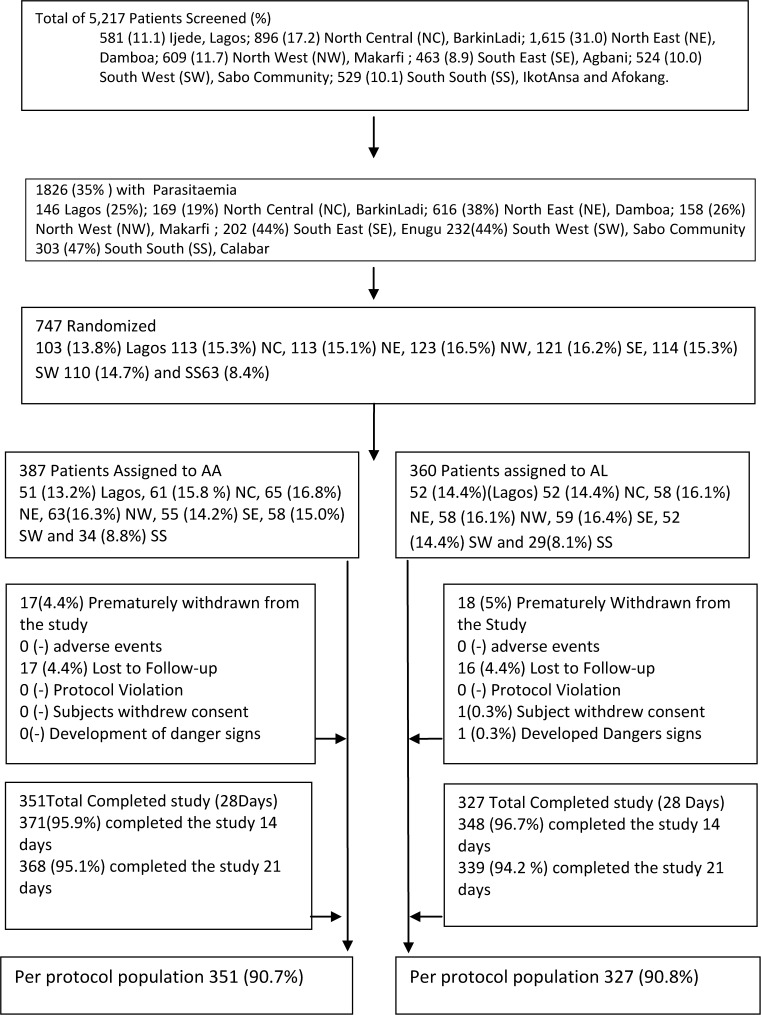
The study profile.

Between October 2009 and November 2010, 747 patients were recruited: 360 in artemether-lumefantrine and 387 in artesunate-amodiaquine groups (Figure 2

). Eighteen and 17 children were withdrawn prematurely in the artemether-lumefantrine and artesunate-amodiaquine groups, respectively, during the first week of follow-up. The baseline characteristics were similar for both treatment groups (Table 1). However, the number of children < 1 year of age was significantly lower in the artesunate-amodiaquine compared with the artemether-lumefantrine treatment arms.

### Primary outcomes.

#### PCR-uncorrected and PCR-corrected efficacy estimates (including time to recrudescence and new infections).

Overall, ACPR in both treatment groups on Days 14, 21, and 28 was seen in 713 of 719 children (99.2%, 95% confidence interval [CI] 98.1–99.7%), 681 of 707 children (96.3%, 95% CI 94.6–97.5%), and 658 of 678 children (96.3%, 95% CI 94.4–97.6%), respectively. In children treated with artemether-lumefantrine, ACPR on Days 14, 21, and 28 was seen in 344 of 348 children (98.9%, 95% CI 96.9–99.6%), 329 of 339 children (97.1%, 95% CI 94.5–98.5%), and 316 of /327 children (95.1%, 95% CI 92.0–97.1%), respectively. In children treated with artesunate-amodiaquine, ACPR on Days 14, 21, and 28, was seen in 369 of 371 children (99.5%, 95% CI 97.9–99.9), 352 of 368 children (95.7%, 95% CI 92.9–97.4%), and 342 of 351 children (97.4%, 95% CI 95.0–98.7%), respectively. There were no significant differences in these parameters at all times in both treatment groups (*P* = 0.63, 0.32, 0.16, on Days 14, 21, and 28, respectively). Response to both treatment regimens was not related to age: 13 of 123 and 18 of 236 of ≤ 2 and > 2 years of age, respectively, treated with artemether-lumefantrine failed treatment by Day 28 (*P* = 0.46). Similarly, 10 of 114 and 18 of 271 ≤ 2 and > 2 years of age, respectively, treated with artesunate-amodiaquine failed treatment by Day 28 (*P* = 0.60).

#### Recrudescent and new infections.

Of the 72 recurrent infections during the 28-day follow-up period, 21 were new infections and 34 were recrudescent infections of *P. falciparum.* In 17 cases PCR results were not available, that is, unknown. Overall, the PCR corrected parasitologic cure rates on Days 14, 21, and 28 were 713 of 719 (99.2%, 95% CI 98.1–99.7%; 689 of 707, 97.5%, 95% CI 95.9–98.4%; 661 of 678, 97.5%, 95% CI 95.9–98.5%). In artemether-lumefantrine-treated children, PCR corrected parasitologic cure rates on Days 14, 21, and 28 were 344 of 348, 98.9%, 95% CI 96.9–99.6%; 333 of 339, 98.2%, 95% CI 96.0–99.3%; and 316 of 327, 96.6%, 95% CI 93.9–98.2%, respectively. In artesunate-amodiaquine-treated children, PCR corrected parasitologic cure rates on Days 14, 21, and 28 were 369 of 371, 99.5%, 95% CI 97.9–99.9%; 356 of 368, 96.7%, 95% CI 94.2–98.2%; and 345 of 351, 98.3%, 95% CI 96.1–99.3%, respectively. There were no significant differences in these parameters at all times in both treatment groups (*P* = 0.63, 0.21, 0.26, on Days 14, 21, and 28, respectively).

**Figure 3.**
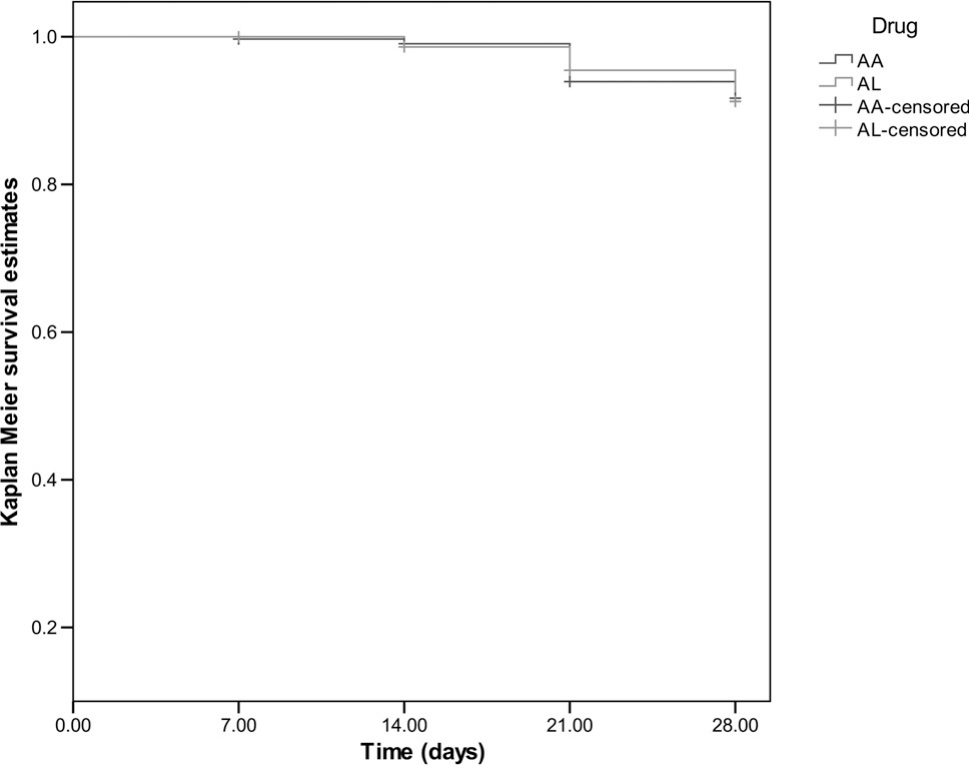
Kaplan-Meier survival estimates of asexual parasitemia after treatment with artesunate-amodiaquine (AA, blue solid line) or artemether-lumefantrine (AL, green broken line). Log-rank statistic = 0.027, *P* = 0.869). Pooled data from all sentinel sites.

The median times to recrudescence and new infections (reinfections) were similar (21 days, range from 7 to 28 days versus 21 days, range from 7 to 28 days). Recurrent infections were similar in both treatment groups: artemether-lumefantrine, 24 of 349; artesunate-amodiaquine, 21 of 375, *P* = 0.48). Overall, the probability of reappearance of asexual parasitemia after treatment with both combinations were similar (Log-rank statistic = 0.027, *P* = 0.869, Figure 3). The probabilities of recurrent infections with artesunate-amodiaquine or artemether-lumefantrine were similar at all sites (data not shown for individual sites). Recurrent infections were not related to age: 4 and 68 infections were in the < 1 and = 1 year olds, respectively (4 of 45 versus 68 of 679, *P* = 1.0). Similarly, recrudescent infections were not related to age: 1 and 37 infections were in the < 1 and = 1 year olds, respectively (1 of 44 versus 37 of 667, *P* = 0.5).

In children treated with artemether-lumefantrine, the median dose of lumefantrine was significantly higher in children without recrudescence than in those with recrudescence: 75.8 ([mean 77.2, range 49.7–144] mg/kg, *N* = 314) versus 60 (mean 62.6, range 51.4–90 mg/kg, *N* = 17, *P* = 0.001 by Kruskal Wallis test). The median dose of artemether was also significantly higher in children without recrudescence 12.6 (mean 12.9, range 8.3–24 mg/kg, *N* = 314) versus 10.0 (mean 10.4 range 8.6–15 mg/kg, *N* = 17, *P* = 0.001 by Kruskal Wallis test). In children treated with artesunate-amodiaquine, the median dose of amodiaquine was similar in children without recrudescence and in those with recrudescence: 31.2 (mean 32.8, range 22.8–45 mg/kg, *N* = 345) versus 31.2 (mean 33.3, range 23.8–45 mg/kg, *N* = 17, *P* = 0.78 by Kruskal Wallis test). The median dose of artesunate was also similar in children without and with recrudescence 11.5 (mean 12.2, range 8.4–16.7 mg/kg, *N* = 345), versus 11.5 (mean 12.3, range 8.8–16.7, *N* = 17, *P* = 0.79 by Kruskal Wallis test). The findings on the median dose in the two treatment groups were seen at all study sites (data not shown).

Overall, parasite positivity rate on Day 1 was significantly higher in children with recrudesce compared with those without recrudescence (24 of 33 [72.7%] versus 357 of 665 ([3%], χ^2^ = 4.59, *P* = 0.03 by Mantel-Haenszel test). However, parasite positivity rate on Day 2 were similar in children with recrudesce and those without recrudescence (3 of 34 [8.8%] versus 33 of 666 [5%], *P* = 0.41). In children treated with artemether-lumefantrine, parasite positivity rate on Day 1 were similar in children with recrudesce and those without recrudescence (13 of 16 [81.3%] versus 188 of 319 [58.9%], χ^2^ = 3.15, *P* = 0.076). Parasite positivity rate on Day 2 were also similar in children with recrudesce and those without recrudescence (2 of 17 [11.8%] versus 18 of 319 [5.6%], *P* = 0.26). In children treated with artesunate-amodiaquine, parasite positivity rate on Day 1 were similar in children with recrudesce and those without recrudescence (11 of 17 [64.7%] versus 169 of 346 [48.8%], *P* = 0.20). Parasite positivity rate on Day 2 were also similar in children with recrudesce and those without recrudescence (1 of 17 [5.9%] versus 15 of 347 [4.3%], *P* = 0.54). Parasite positivity rate on Day 3 was also similar in children with recrudescence and in those without recrudescence (1 of 17 [5.9%] versus 3 of 347 [0.9%], *P* = 0.17).

#### Rate of recrudescent infections.

**Figure 4.**
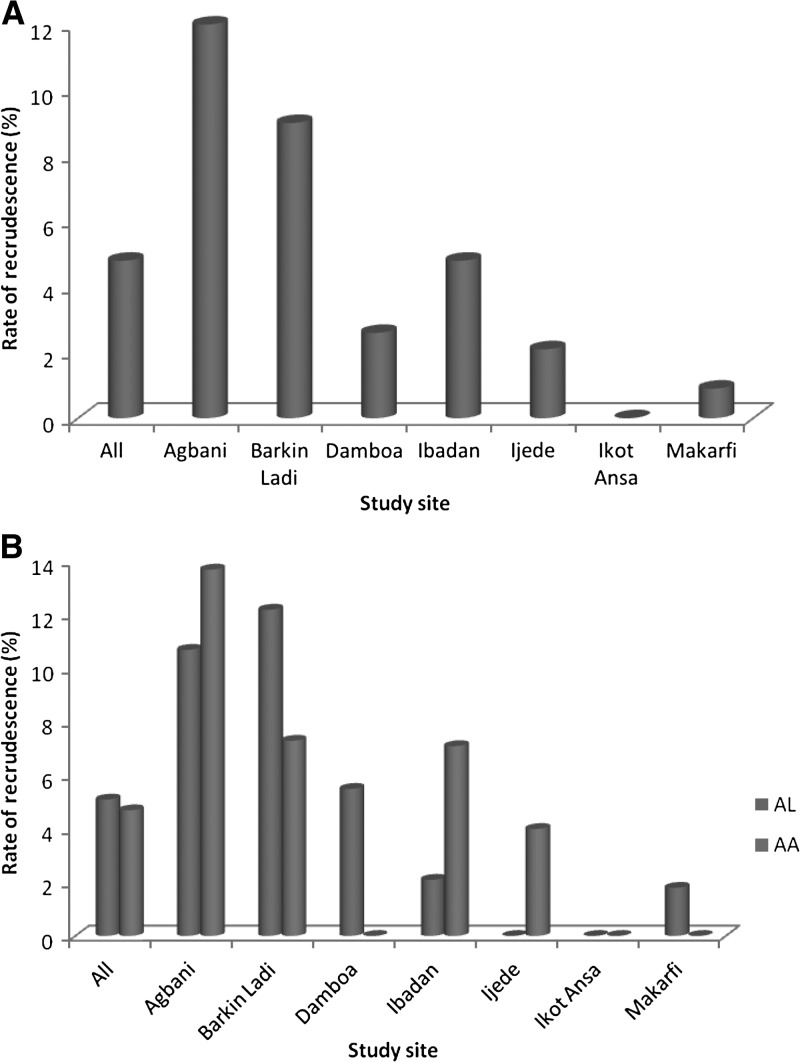
Rate of recrudescence in children with falciparum malaria treated with artemether-lumefantrine or artesunate-amodiaquine (**A**, both ACTs; **B**, individual ACT).

The overall rate of recrudescent infections was 4.8% (34 of 711 children) and was significantly higher at sites on the eastern than on the western flanks 26 of 311 versus 8 of 319, (*P* = 0.002, Figure 4

). Table 2 shows the details of the treatment failures and the recrudescent infections, according to drug treatment at each site. In a multiple logistic regression model, the likelihood of achieving adequate clinical and parasitological response was similar using artemether-lumefantrine and artesunate-amodiaquine (OR = 0.78, 95% CI 0.36–1.68, *P* = 0.52). However, treatment outcome was more likely to be different in Agbani (Enugu State, OR = 0.2, 95% CI 0.05–0.7, *P* = 0.01) and Barkin Ladi (Plateau State, OR = 0.25, 95% CI 0.07–0.94, *P* = 0.04) for both drugs if adequate clinical and parasitological response in Damboa (Borno State) or other sites was used as the reference. Recrudescent infections were not related to enrollment parasitemia: 4 of 71 and 31 of 675 children with a parasitemia ≥ 100,000/μL and < 100,000/μL, respectively, had a PCR-confirmed recrudescence (*P* = 0.52); geometric mean parasitemia (recrudescent versus non-recrudescent 19,328 versus 15,355, *P* = 0.68; age: 37.1 ± 14.9 versus 36.3 ± 16.3 months, *P* = 0.77; enrollment body temperature: 38.0 ± 1.1 versus 38.0 ± 1.1°C, *P* = 0.69; or gender (proportion of female): 14 of 35 versus 296 of 274, *P* = 0.77.

#### Parasite positivity rates on Day 3 and parasite clearance.

Overall, at enrollment, 675 of 727 children (93%) had a parasitemia < 100,000/μL: 322 and 353 children in artemether-lumefantrine and artesunate-amodiaquine treatment arms, respectively. In these 675 children, parasitemia on Day 3 was seen in 5 children: 2 in artemether-lumefantrine and 3 in artesunate-amodiaquine-treated children—an overall parasite positivity rate on Day 3 of 0.7%. The five patients with parasitemia on Day 3, were scattered among five different study sites. Patient enrollment at each of these study sites was ∼50–60 patients per treatment arm. Thus, there was no evidence of any cluster of cases with slow parasite clearance after treatment.^19^

Clearance of parasitemia was evaluated in all children who completed a minimum follow-up of 7 days (*N* = 724): 349 children treated with artemether-lumefantrine and 375 children treated with artesunate-amodiaquine). By Day 1, parasitemia cleared in 46% of the children (*N* = 724), including 41% (*N* = 349) of the children treated with artemether-lumefantrine and in 50% (*N* = 375) of the children treated with artesunate-amodiaquine. The difference between these two proportions was statistically significant (*P* = 0.013). By Day 2, parasitemia was still present in 6% of children treated with artemether-lumefantrine and in 4.5% of children treated with artesunate-amodiaquine. The difference between these two proportions was not statistically significant (*P* = 0.47). Overall, parasite clearance times were similar with both artemether-lumefantrine and artesunate-amodiaquine treatments (1.11 ± 0.34, range 1–3 days 95% CI 1.07–1.15, versus 1.13 ± 0.4, range 1–3 days 95% CI 1.09–1.17, *P* = 0.47). Parasite clearance times were similar in the two treatment arms at all study sites (data not shown). In one 34-month-old child, treated with artesunate-amodiaquine, parasitemia persisted until Day 7 of follow-up. The child was hyperparasitemic at enrollment (parasitemia was 150,000/μL). No patient received rescue medication.

Parasite clearance was significantly longer in children with recrudescent infections compared with those without recrudescence (1.8 + 0.5 d, range 1–3, *N* = 32 versus 1.6 + 0.7 d, range 1–7, *N* = 587, *P* = 0.019). However, fever clearance was similar in children with and without recrudescence (1.04 + 0.2 d, range 1–2, *N* = 23 versus 1.13 + 0.5 d, range 1–7, *N* = 440, *P* = 0.42). The mean weights in children with and without recrudescence were similar (14.6 + 3.8 kg, range 9–24, *N* = 34. versus 0.13.3 + 4.3 d, range 5.5–24, *N* = 663, *P* = 0.07).

### Secondary outcomes.

#### Fever clearance.

Five hundred and forty children were febrile at enrollment: 258 of 349 in the artemether-lumefantrine and 282 of 375 in the artesunate-amodiaquine groups. By Day 1, fever had cleared in 227 children (88%) treated with artemether-lumefantrine and in 260 children (92%) treated with artesunate-amodiaquine. The difference between these proportions was not statistically significant (*P* = 0.10). By Day 2, fever had cleared in 28 children treated with artemether-lumefantrine and in 19 children treated with artesunate-amodiaquine. The difference between these proportions was also not statistically significant (*P* = 0.09). Overall, fever clearance was significantly faster with artesunate-amodiaquine compared with artemether-lumefantrine-treated children (1.19 ± 0.5, range 1–3, 95% CI 0.82–1.56 versus 1.33 ± 0.7, range 1–3, 95% CI 0.81–1.85 days, *P* = 0.006). At all sites fever clearance times were similar in artemether-lumefantrine-treated children (data not shown). Similarly, at all sites fever clearance times were similar in artesunate-amodiaquine-treated children (data not shown).

#### Gametocyte carriage.

Gametocytes were detected in the peripheral blood of 159 of 623 children (25.5%) from the two treatment groups (Table 3). The detection rate at enrollment was not significantly different between the two treatment groups (*P* = 0.36). Overall, post-treatment gametocyte carriage was significantly lower than pretreatment gametocyte carriage in both treatment groups (*P* < 0.0001). Overall, baseline gametocyte carriage was significantly higher in children with recrudescent infections than in those without recrudescence (15 of 31 children [48.4%] versus 133 of 554 children [24%], *P* = 0.002). On Day 14, gametocytes were not seen in peripheral blood of all children with recrudescent infections (*N* = 34) but were detectable in the peripheral blood in 1.3% of those without recrudescence (*N* = 553). In children treated with artemether lumefantrine, 50% (*N* = 14) of those with recrudescence had gametocytes at baseline, but gametocytes were not detectable in the peripheral blood of these children on Day 14 (*N* = 13).

#### Recovery from malaria-associated anemia.

**Figure 5.**
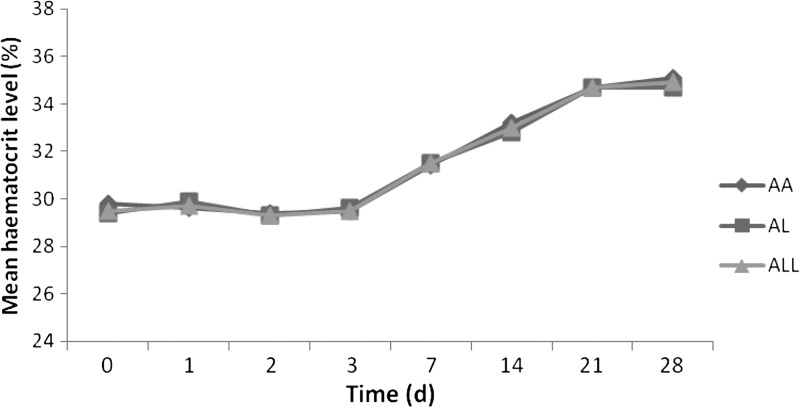
Changes in hematocrit before, during, and after treatment with artemether-lumefantrine (AL) or artesunate-amodiaquine (AA) in children with falciparum malaria.

Hematocrit data were available in 672 children at enrollment and in 646 children 28 days after enrollment (Table 4). Anemia (hematocrit < 30%) was present in 294 children (44%) at enrollment and was not significantly different between the two treatment groups (*P* = 0.87). After treatment, the prevalence of anemia was similar in both treatment groups (*P* = 0.71). Post-treatment prevalence of anemia was significantly lower than pretreatment in both treatment groups (*P* < 0.0001). Only 2% of children (12 of 646) who were anemic at enrollment were still anemic by Day 28. Changes in hematocrit before, during, and after treatment with both treatment regimens were similar (Figure 5

). In 272 patients who were anemic at enrollment, mean times to attainment of a hematocrit value ≥ 30% (recovery time) was 10 days (range 1–28 days, 95% CI 9.04–10.9, *N* = 272), and was similar in artemether-lumefantrine- and artesunate-amodiaquine-treated children (mean 10.5 days, range 1–28 days, 95% CI 9.2–11.8, *N* = 135 versus mean 9.5 days, range 1–28 days, 95% CI 8.1–10.8, *N* = 137, *P* = 0.17). Anemia recovery time was not related to age: in ≤ 2 and > 2 years of age, mean times were 11.3 days, range 1–28, 95% CI 9.6–12.9, *N* = 92, and 9.3 days, range 1–28 days, 95% CI 8.2–10.4, *N* = 179, respectively, *P* = 0.53). Overall, anemia recovery time was significantly longer in children with enrollment parasitemia > 50,000/μL than in children with enrollment parasitemia ≤ 50,000/μL (14.2 days, range 7–28 days, 95% CI 12.6–15.8, *N* = 52 versus 12.3 days, range 1–28 days, 95% CI 11.5–13.2, *N* = 222, *P* = 0.015, by Kruskal-Wallis test). In children treated with artesunate-amodiaquine, anemia recovery time was also significantly longer in children with enrollment parasitemia > 50,000/μL than in children with enrollment parasitemia ≤ 50,000/μL (14.8 days, range 7–28 days, 95% CI 12.7–16.9, *N* = 25 versus 11.9 days, range 1–28 days, 95% CI 10.8–13.1, *N* = 116, *P* = 0.008). In children treated with artemether-lumefantrine, anemia recovery times were similar in the children with enrollment parasitemia > 50,000/μL and those with enrollment parasitemia ≤ 50,000/μL (13.7 days, range 7–28 days, 95% CI 11.4–16.1, *N* = 27 versus 12.8 days, range 7–28 days, 95% CI 11.5–14.1, *N* = 106, *P* = 0.39).

In 289 anemic children (from both treatment arms) in whom hematocrit data were available at enrollment and 28 days after enrollment, hematocrit was significantly higher after treatment than before treatment (33.3 ± 4.2 versus 25.2 ± 3.4%, *P* < 0.0001). In 142 anemic children treated with artemether-lumefantrine, hematocrit was also significantly higher after treatment than before treatment (33.0 ± 4.1 versus 25.2 ± 3.3%, *P* < 0.0001). Similarly, in 147 anemic children treated with artesunate-amodiaquine, hematocrit was also significantly higher after treatment than before treatment (33.4 ± 4.3 versus 25.3 ± 3.6%, *P* < 0.0001).

### Adverse events.

Adverse events were carefully documented at two sites: Barkin Ladi and Sabo Quarters of Ibadan. Overall 78 of 223 children (32 of 104 in artemether-lumefantrine and 46 of 119 in artesunate-amodiaquine) reported at least one adverse event within the first week of starting treatment. There was no significant difference in the proportions of patients reporting adverse events in both treatment groups (*P* = 0.28). Seven children (3 in artemether-lumefantrine and 4 in artesunate-amodiaquine) reported more than two adverse events. Many of the children reporting adverse events were > 2 years of age. Table 5 is a summary of adverse events reported within the first week. In both treatment groups, cough was the most frequently reported adverse event. There were no significant differences in the frequencies of the reported adverse events in both treatment groups.

## Discussion

In this relatively large study conducted at sites representing most geographical areas of Nigeria, and at ∼5 years after adoption of artemether-lumefantrine and artesunate-amodiaquine as first-line treatments,^10^ both treatment regimens proved to be effective treatments for acute, uncomplicated *P. falciparum* malaria in children < 5 years of age. It is noteworthy that despite high grade chloroquine resistance and the association between transporter genes and resistance to chloroquine or amodiaquine in the area,^20^–^24^ artesunate-amodiaquine was still effective in the children < 5 years of age. The latter may be a result of the very limited use of amodiaquine monotherapy before the adoption of artesunate-amodiaquine as one of the first-line antimalarial drugs.^10^,^25^,^26^ and the fact that amodiaquine is more effective even in areas with considerable chloroquine resistance.^27^ The relatively low prevalence (7%) in Nigerian isolates of *Pfcrt* (CVMNT) haplotype^28^ and possibly of other haplotypes of *Pfmdr 1* and *Pfcrt* (SVMNT),^29^,^30^ may also be contributory.

As anticipated, fever clearance was significantly faster in children treated with artesunate-amodiaquine compared with those treated with artemether-lumefantrine. This may be caused by the antipyretic effect of the amodiaquine component of artesunate-amodiaquine.^24^,^27^ It is generally thought that the lumefantrine component of artemether-lumefantrine has little or no significant antipyretic effect. Compared with artemether-lumefantrine, artesunate-amodiaquine also reduced significantly the proportion of children with parasitemia 1 day after treatment began. This is in line with a recent study from southwestern Nigeria evaluating the therapeutic efficacy of both regimens in the first 5 years of adoption in Nigeria.^26^ However, parasite clearance times were similar in both treatment groups. The reasons for the artesunate-amodiaquine significantly reducing the proportion of children with parasitemia are not apparent from the results of this study. Perhaps, amodiaquine in artesunate-amodiaquine complemented the rapid reduction in parasitemia by artesunate 1 day after treatment began significantly more than lumefantrine complemented the effects of artemether 1 day after treatment began. It is also possible that differential rates of absorption and bioavailability of the two artemisinin derivatives may be contributory.^31^

The significantly reduced gametocyte carriage post treatment by the two ACTs is in keeping with previous reports.^1^,^25^,^26^ An important finding is the lower dose of lumefantrine in patients with recrudescent infections when compared with patients without recrudescent infections. This outcome is unlikely to be a result of *in vivo* resistance of *P. falciparum* to lumefantrine in children who received the lowest dose of lumefantrine, because no previous studies in Nigeria have shown that such lower doses of lumefantrine resulted in clearance of parasitemia in the region, that would indicate a change in susceptibility to antimalarial drugs.^32^

Based on the 3% threshold of parasite positivity on Day 3 defined by Stepniewaska and others,^19^ with a sample size of at least 50 patients, there is no indication of reduced *in vivo* sensitivity to artemisinin in the different regions of Nigeria 5 years after adoption of artemisinin-based combination treatments. The evidences to support the latter are: overall efficacy of 96.5%^26^ for both artemisinin-based combination treatments and an overall PCR-corrected cure rate on Day 28 of 97.5% for both artemisinin-based combination treatments in the present study.

Recrudescent infections occurred in 5% of the children and the rates were significantly higher at sites located on the eastern than on the western flank (Figure 4). The reasons for the difference in rates are unclear. However, it may not be unrelated to the historical pattern of reduced susceptibility in *P. falciparum* to antimalarial drugs in Nigeria. For example, chloroquine or sulfadoxine-pyrimethamine resistance was more frequently reported in the eastern than in the western region of the country.^33^ Recrudescent infections in the cohort of children evaluated was not related to age, gender, or parasite density at enrollment. In Thailand, before the emergence of resistance to artemisinin, recrudescent infections were not indicative of resistance to artemisinin drugs, and were thought to be related to the relatively short half-lives of these drugs.^34^ However, with the emergence of resistance to artemisinin on the western boarder of Thailand, resistance to artemisinin has been related to both density of parasitemia and parasite genotypes.^7^ Therefore, when *P. falciparum* strains with reduced susceptibility to artemisinin will emerge or spread to Nigeria, a positive relationship should be found between parasite density and recrudescence of infections in endemic areas of Nigeria.

Recent reports indicate that resistance in *P. falciparum* has emerged to artemisinin in Southeast Asia, particularly in Cambodia, Thai-Cambodian border and the western border of Thailand where artemisinin combination drugs have been deployed,^3^–^7^,^35^,^36^ and has been linked to changes in chromosome 13 of the parasite.^37^ There is currently no evidence of the development and spread of artemisinin-resistant parasites outside the greater Mekong Subregion. Although the standard *in vitro* test is not reliable for artemisinin compounds, and new tests are being developed,^38^ previous reports of reduced susceptibility of *P. falciparum* isolates *in vitro* to artemisinin drugs in Nigeria^39^,^40^ would justify the need for frequent evaluation and close monitoring of the sensitivity of *P. falciparum* isolates *in vivo*, and *in vitro*, and by molecular markers in endemic areas of Nigeria. The recent identification of molecular markers will help in monitoring the distribution and spread of resistance and to understanding the evolution and mechanism(s) of resistance in *P. falciparum* to artemisinin drugs.^41^ Evaluation of the time course of change in parasitemia half-life may also contribute to early detection of development of artemisinin resistance.^7^,^26^ In this context, the parasitemia half-life of 1 hour after treatment with artemether-lumefantrine and artesunate-amodiaquine recently reported in this endemic area^26^ is considerably lower than from Vietnam 8 hours^42^ or Thailand 3 hours^19^ and western border of Thailand where the half-life lengthened from a mean of 2.6 hours in 2001 to 3.7 hours in 2010,^7^ to a mean of 5.5 hours in western Cambodia^7^—areas where artemisinin resistance has been reported after deployment of artemisinin-based combination treatments.

Compared with non-artemisinin or non- artemisinin-based combination treatments, artemisinin drugs and artemisinin-based combination treatments may retard the elimination, by the spleen, of *P. falciparum-*infected red blood cells, particularly when parasitemias are high.^43^,^44^ Although the circulating life span of these infected red blood cells from which the parasites have been removed by pitting may be short,^45^ precipitous falls in hematocrit may be prevented in the first week after treatment.^44^ The relatively short anemia recovery time of ∼10 days in the children evaluated, is in line with previous study showing a prompt hematological recovery after ACTs.^44^ The significantly lower prevalence of anemia post-treatment compared with pretreatment, and the similar anemia recovery times in both artesunate-amodiaquine- and artemether-lumefantrine-treated children suggest that both regimens are equivalent in their effects on malaria-associated anemia in children. However, in other studies, anemia has been shown to be more frequent and the average rate of hemoglobin 7 days after treatment began, lower in patients treated with artesunate-amodiaquine compared with those treated with artemether-lumefantrine.^46^ Additionally, Zwang and others^47^ have shown that the proportion of patients recovering from anemia 28 days after treatment began was significantly lower in patients treated with artesunate-amodiaquine compared with amodiaquine alone. The reasons for the significantly increased anemia recovery time in patients with parasitemia in excess of 50,000/μL of blood compared with those with parasitemia < 50,000/μL of blood who were treated with artesunate-amodiaquine are not immediately apparent in this cohort of children. This would require further evaluation in children in this endemic area after treatment with ACTs.

Reported adverse events to both treatment regimens were similar, did not necessitate discontinuation of treatment in any child, and were, in general, indistinguishable from the symptoms and signs of acute malaria infections. It is noteworthy that no child reported pruritus, an adverse event frequently encountered in Nigerian children treated with 4-aminoquinolines (chloroquine or amodiaquine), and to a lesser extent, halofantrine and mefloquine.^48^ Because lumefantrine is structurally related to halofantrine, it is possible that pruritus may occur in children treated with lumefantrine. Indeed, pruritus has been reported in Nigerian children treated with artemether-lumefantrine.^49^ Additionally, recent study from Liberia has shown that artemether-lumefantrine is better tolerated than artesunate-amodiaquine by the older children and adults.^50^ However, except for fatigue, both ACTs were equally tolerated by children < five years of age.^50^ In conclusion, 5 years after adoption as first-line treatments, artemether-lumefantrine and artesunate-amodiaquine remain efficacious treatments of uncomplicated falciparum malaria in Nigerian children < 5 years of age.

## Figures and Tables

**Table 1:** Baseline characteristics at enrollment

	AL (*N* = 360)	AA (*N* = 387)	All (*N* = 747)	*P* value
Male gender n (%)	201(56)	213(56)	414(56)	0.95
Mean Age (months) (SD)	36.1(17.2)	36.6 (15.2)	36.3 (16.2)	0.83
Number aged < 1 year	28 (7.8%)	17 (4.4%)	45 (6.1%)	0.04
Duration of illness (days)				
Mean (SD)	2.9 (0.9)	3.0(1.3)	3.0(1.1)	0.39
Range	1–6	1–7	1–7	
Weight [Kg]				
Mean (SD)	13.2 (4.5)	13.5 (4.0)	13.3 (4.3)	0.17
Range	5.5–24	5.5–24	5.5–24	
Body temperature (°C)				
Mean (SD)	38.0 (1.1)	38.0 (1.1)	38.0 (1.1)	0.71
Range	35.7–40.5	35.2–40.4	35.2 40.5	
No. with temperature *P* > 37.4 (°C) [%]	267[74.2]	291 [75.2]	558[74.7]	0.81
No. with temperature > 40 (°C)	13 (3.6)	12 (3.1)	25 (3.3)	0.85
Hematocrit				
Mean (SD)	29.4 (5.3)	30.0 (5.7)	29.7 (5.5)	0.34
Range	17–42	15–43	15–43	
No. with hematocrit < 30%	68(*N* = 154)	76(*N* = 169)	144(*N* = 323)	0.88
Geometric mean parasitemia (/μL) and range	15169 (1,290–200,000)	14370 (1,100–200,000)	14749 (1,100–200,000)	0.60
Number with gametocytaemia (%)	82 (22.8)	77 (19.9)	159 (21)	0.63

AL = artemether-lumefantrine; AA = artesunate-amodiaquine.

Liver was enlarged in 161 of 517 children (31.1%) and spleen in 135 of 517 children (26.1%), *P* = 0.07.

**Table 2:** Efficacy of artemether-lumefantrine or amodiaquine-artesunate according to study site*

Artemether-lumefantrine:							
State	PCR uncorrected			PCR corrected			
	ACPR_u	Failure_u	Total	ACPR_c	Recrudescence	Total	% Recrudescence
Borno (Damboa)	51	5	56	52	3	55	5.5
Cross River (Ikot Ansa)	26	1	27	27	0	27	0
Enugu (Agbani)	48	8	56	50	6	56	10.7
Kaduna (Makarfi)	55	1	56	55	1	56	1.8
Lagos (Ijede)	43	4	47	45	0	45	−
Oyo (Ibadan)	47	1	48	47	1	48	2.1
Plateau (Barkin Ladi)	41	11	52	43	6	49	12.2
Total	311	31	342	319	17	336	5.1
Amodiaquine-artesunate:							
State	PCR uncorrected			PCR corrected			
	ACPR_u	Failure_u	Total	ACPR_c	Recrudescence	Total	% Recrudescence
Borno (Damboa)	58	2	60	60	0	60	−
Cross River (Ikot Ansa)	33	1	34	34	0	34	−
Enugu (Agbani)	44	8	52	44	7	51	13.7
Kaduna (Makarfi)	59	0	59	59	0	59	−
Lagos (Ijede)	48	2	50	48	2	50	4.0
Oyo (Ibadan)	52	4	56	52	4	56	7.1
Plateau (Barkin Ladi)	48	11	59	51	4	55	7.3
Total	342	28	370	348	17	365	4.7

* ACPR u = adequate clinical and parasitological response uncorrected. ACPR c = adequate clinical and parasitological response corrected. Failure u = treatment failure uncorrected; PCR = polymerase chain reaction.

**Table 3:** Gametocyte carriage before and after treatment of malarious children with artesunate-amodiaquine or artemether-lumefantrine*

	No. with gametocytemia		*P* value
	Before treatment	After t	
AA	77/321	2/298	< 0.0001
AL	82/302	0/281	−
Total	159/623	2/579	< 0.0001

* AL = artemether-lumefantrine; AA = artesunate-amodiaquine.

**Table 4:** Malaria-associated anemia before and after treatment with artemether-lumefantrine or artesunate-amodiaquine

	Anemia*		*P* value
	Before treatment (D0)	After treatment (D28)	
AL	152/345	5/331	< 0.00001
AA	142/327	7/315	< 0.00001
Total	294/672	12/646	< 0.00001

* Hematocrit < 30%.

AL = Artemether-lumefantrine; AA = Artesunate-amodiaquine.

**Table 5:** Adverse events reported within the first week of the study*

	AL	AA
Number of children	104	119
Fever	6	7
Vomiting	2	8
Abdominal pain	5	7
Weakness	0	2
Headache	5	4
Anorexia	5	8
Puffy face	1	2
Abdominal distension	1	1
Cough	14	16

* Data from two sites.

AL = Artemether-Lumefantrine; AA = Artesunate-Amodiaquine.
